# The Relationship Between Financial Well-Being and Antiretroviral Therapy Adherence in Youth with HIV in the United States

**DOI:** 10.1007/s10461-026-05145-y

**Published:** 2026-04-28

**Authors:** Marie C. D. Stoner, Isha Shrestha, Torsten B. Neilands, Kristin Ming, Louis Smith, Celeste Balaban, Mallory O. Johnson, Parya Saberi

**Affiliations:** 1 RTI International, Research Triangle Park, NC USA; 2 Center for AIDS Prevention Studies, University of California, San Francisco, CA USA; 3 University of Washington School of Medicine, Seattle, WA USA

**Keywords:** Financial well-being, Subsistence needs, Antiretroviral therapy adherence, Youth with HIV, Long-acting injectables

## Abstract

Youth and young adults experience high attrition across the HIV care continuum, including elevated risk of antiretroviral therapy (ART) nonadherence and virologic failure. This study examined how financial well-being relates to ART adherence among youth with HIV (YWH), including those using oral or LAI-based regimens. We analyzed baseline data from YWH aged 18–29 years in the United States enrolled between 2023 and 2025 in a randomized controlled trial addressing HIV care barriers, mental health, and substance use. Oral ART adherence was measured using a validated scale, with high adherence defined as a score ≥ 80%. For participants on LAI-ART, high adherence was defined as having no missed or delayed injection visits. We assessed the associations between financial well-being (i.e., unmet subsistence needs, overall financial situation, and mobile technology vulnerability) and adherence using descriptive statistics and adjusted prevalence ratios (PRs) estimated with log-Poisson models with robust standard errors. Among 201 participants, the median age was 27 years. Most (89.1%, *n* = 179) used oral ART, while 10.0% (*n* = 20) received LAI-ART. High adherence was achieved by 69.8% of oral ART users and 90% of LAI-ART users. Participants who reported they could “barely get by” financially had significantly lower adherence compared to those living comfortably (RR 0.70, 95% CI 0.52–0.95). Greater unmet subsistence needs were also associated with reduced adherence (RR 0.85, 95% CI 0.73–0.99). Financial well-being was linked to adherence among YWH using both oral and LAI-ART. Efforts to reduce financial hardship may support improved HIV treatment outcomes.

## Resumen

Los adolescentes y adultos jóvenes presentan una alta pérdida de seguimiento a lo largo del continuo de atención del VIH, incluyendo un riesgo elevado de falta de adherencia a la terapia antirretroviral (TAR) y de fracaso virológico. Este estudio examinó la relación entre el bienestar financiero y la adherencia a la TAR entre jóvenes con VIH (JcVIH), incluyendo aquellos que utilizan regímenes orales o basados en inyecciones de acción prolongada (LAI). Se analizaron datos basales de JcVIH de 18 a 29 años en Estados Unidos, inscritos entre 2023 y 2025 en un ensayo controlado aleatorizado que aborda barreras en la atención del VIH, salud mental y consumo de sustancias. La adherencia a la TAR oral se midió mediante una escala validada, definiéndose alta adherencia como una puntuación ≥ 80%. Para los participantes en TAR-LAI, la alta adherencia se definió como no haber perdido ni retrasado visitas para la administración de inyecciones. Se evaluaron las asociaciones entre el bienestar financiero —necesidades básicas insatisfechas, situación financiera general y vulnerabilidad en el acceso a tecnología móvil— y la adherencia mediante estadísticas descriptivas y razones de prevalencia (RP) ajustadas, estimadas con modelos log-Poisson con errores estándar robustos.Entre 201 participantes, la mediana de edad fue de 27 años. La mayoría (89,1%, *n* = 179) utilizaba TAR oral, mientras que el 10,0% (*n* = 20) recibía TAR-LAI. La alta adherencia se observó en el 69,8% de los usuarios de TAR oral y en el 90% de los usuarios de TAR-LAI. Los participantes que reportaron que “apenas lograban cubrir sus gastos” presentaron una adherencia significativamente menor en comparación con aquellos que vivían cómodamente (RR 0,70; IC 95%: 0,52–0,95). Un mayor número de necesidades básicas insatisfechas también se asoció con una menor adherencia (RR 0,85; IC 95%: 0,73–0,99).El bienestar financiero se asoció con la adherencia entre JcVIH que utilizan tanto TAR oral como TAR-LAI. Las intervenciones dirigidas a reducir las dificultades económicas podrían contribuir a mejorar los resultados del tratamiento del VIH.

## Introduction

 Youth with HIV (YWH) face significant challenges in maintaining engagement across the HIV care continuum, often experiencing higher rates of antiretroviral therapy (ART) nonadherence and subsequent virologic failure compared to older populations [1–4]. This demographic is particularly vulnerable due to developmental, psychosocial, and structural factors that impede consistent healthcare engagement [5–8]. Financial well-being, encompassing factors such as financial stability and the ability to meet basic subsistence needs (e.g., food, water, shelter, and clothing), plays a critical role in health outcomes for individuals with chronic conditions, including HIV [9–13]. Financial hardships can lead to decreased healthcare engagement, medication nonadherence, and poorer health outcomes. Understanding the relationship between financial well-being and ART adherence is crucial for developing targeted interventions that address these socioeconomic barriers, particularly as new HIV prevention methods become available.

Long-acting injectable ART (LAI-ART; injections every 4 or 8 weeks) has emerged as a promising alternative to daily oral regimens, potentially enhancing adherence by reducing the burden of daily medication management. Studies have demonstrated that LAI-ART can improve adherence rates among YWH, addressing some of the challenges associated with daily oral ART [14–16]. However, despite the clinical advantages of LAI-ART, structural barriers—such as financial instability, limited access to healthcare facilities, and social determinants of health—continue to impede optimal adherence [15, 17].

This study aims to examine the association between indicators of financial well-being, including housing, unmet subsistence needs, financial stability, and mobile technology vulnerability, with ART adherence among YWH. By identifying the specific financial factors that influence adherence, we can inform the development of comprehensive strategies to support this vulnerable population in achieving optimal health outcomes.

## Methods

This study aimed to examine the relationship between financial well-being and ART adherence among YWH, including those using LAI-ART and daily oral ART. We conducted a cross-sectional analysis using baseline data from YWH aged 18–29 years who were enrolled in the intervention for virologic suppression (iVY) study, a randomized controlled trial addressing HIV care barriers, mental health, and substance use in the United States [18]. iVY enrolled 201 YWH from 2023 to 2025 who were between the ages of 18–29 and resided and received care in California and Florida. Participants were eligible if they had a history of inadequate virological suppression, defined as any HIV viral load ≥ 20 copies/mL in the past 12 months as documented in their clinical records, were in care, had not been recently diagnosed in the last 3 months, had access to a smartphone, and self-identified as English speakers. Individuals with hemophilia and those unable to perform finger pricks at home for the VL test were excluded, as at-home VL testing was required for participation. Any individual displaying extreme mental health or substance use challenges to a degree that impeded the understanding and completion of consent by study staff was excluded and referred to appropriate services. Recruitment was conducted through the AIDS Healthcare Foundation (AHF) sites in Florida and California. AHF contacted patients in their database who met the eligibility criteria. Interested participants were referred to the study team for further screening and enrollment. Additionally, recruitment efforts extended to other non-AHF clinics in California and Florida, including social media outreach and flyers posted at healthcare clinics serving YWH. At enrollment, participants completed an online survey that included questions on demographics, HIV care, ART adherence, and financial stability.

### Measures

For the outcome of ART adherence, participants were asked to self-report “Are you currently taking HIV medications” with response options of “*yes*,* oral pills*,*”* “*yes*,* injections*,” or “*no*.” For participants who reported taking oral ART, adherence was assessed through self-report using a 3-item scale that has been previously validated as a measure of medication adherence by Wilson et al. [19, 20] Responses to these three adherence questions were transformed to a 0–100% scale, with higher scores indicating better adherence. One question was a numeric percentage of days adherent out of 30 days (0–100%). For the two questions that were ordinal, each score was assigned a numeric value (e.g., “*never”=*0, “*rarely”*=20, “*sometimes”*=40, “*usually”*=60, “*almost always*” =80, and “*always”*= 100%; or for other response options: “*Very poor”*=0, “*Poor*”=20%… “*Excellent*”=100%). Scores for the three items were then summed, and the mean of the three individual items was also taken to represent a mean score. High adherence to oral ART was defined as ≥ 80%, consistent with evidence from contemporary ART regimen studies demonstrating viral suppression at adherence levels as low as 75–82%, [9, 21, 22] and with the validation of the adherence measure used in this study at this threshold [19, 20]. Participants who reported using LAI-ART were asked to self-report how frequently they delayed their injection at all in the last 6 months (not at all, a few times, more than half of the time, nearly every time) and how frequently they missed their injection altogether in the last 6 months (not at all, a few times, more than half of the time, nearly every time). Participants who reported any missed or delayed injections were categorized as non-adherent; otherwise, they were considered adherent.

Financial well-being was assessed using self-reported measures of financial situation, housing stability, unmet subsistence needs, and mobile technology vulnerability. The current financial situation was a categorical variable based on self-reported responses to the question “*Which of the following statements best describes your financial situation*?” Response options included “*I have enough money to live comfortably*,” *“I can barely get by on the money I have*,” and “*I cannot get by on the money I have*.” Housing instability was defined as self-report of having “*ever been unhoused*,*” “found yourself without a place to stay for a night or an extended period*,” or “*lived in a shelter*.” Unmet subsistence needs were measured using a 5-item scale previously validated [13, 23]. Participants were asked to respond “yes” or “no” to each item, and responses were summed to create a score of 0–5, with higher scores indicating greater unmet subsistence needs in the past 3 months. Examples of items included “*did you have difficulty finding enough to eat*,” and “*did you have difficulty finding clothing*.” Unmet subsistence needs were examined as a continuous score [13, 23]. Mobile technology vulnerability was a continuous score measured using the Mobile Technology Vulnerability Scale (MTVS) [10]. MTVS is a 17-item scale designed to measure an individual’s sense of stability regarding their access to mobile technology, specifically through their mobile phone. Each item was asked as a “yes” or “no” question regarding the last 6 months. Items included “*at any time in the last 6 months*,* I received formal assistance to pay for my cellphone service*,” and “*at any time in the last 6 months*,* my family or friends helped me to pay for my cell phone service*.” An MTVS score was calculated by the mean of the 17 items [10]. Covariates included state (Florida or California), age in years, sex at birth, race, and ethnicity.

### Statistical Analysis

Descriptive statistics were used to summarize baseline participant characteristics and ART adherence measures. Percentages were reported for binary and categorical measures. Medians with the interquartile range (IQRs) were reported for continuous measures. Bivariate relationships were then examined between measures of financial well-being and adherence to both oral ART and LAI-ART, and only including oral ART. Lastly, we estimated adjusted prevalence ratios (aPRs) and 95% confidence intervals (CIs) for associations between measures of financial well-being and ART adherence using log-Poisson regression models with robust standard errors. Adjusted models accounted for state, age, sex at birth, race, and ethnicity. Models were estimated separately for each financial well-being measure and ART adherence outcomes, once including LAI-ART and once excluding LAI-ART to focus on oral ART only.

## Results

A total of 201 YWH completed their baseline survey from November 29, 2023, to November 2025. The median age of enrolled participants was 27 years (IQR 25–28; Table 1). Most (73.6%; *n* = 73.6%) identified as cisgender male, 7.5% (*n* = 15) as cisgender female, 9.0% (*n* = 18) as non-binary, 4.0% (*n* = 8) as a transgender man or woman, 5.5% (*n* = 11) other gender identity, and 3% (*n* = 6) as unknown. Racially, 37.3% (*n* = 75) identified as Black or African American, 24.4% (*n* = 49) as White, and 47.8% (*n* = 96) as Hispanic or Latino. Approximately two-thirds (67.2%, *n* = 135) of participants were enrolled in California, and 32.8% (*n* = 66) in Florida.

**Table 1:** Baseline demographic and behavioral characteristics (n=201)

	Median (IQR) or n (%)
Age, Median (IQR)	27.0 (25.0, 28.0)
Gender Identity ^1^	
Cisgender Male	148 (73.6%)
Cisgender Female	15 (7.5%)
Non-binary	18 (9.0%)
Transgender man/woman	8 (4.0%)
Other	11 (5.5%)
Unknown Gender Identity	6 (3.0%)
Race	
American Indian / Alaska Native	3 (1.5%)
Asian	3 (1.5%)
Native Hawaiian or Other Pacific Islander	1 (0.5%)
Black or African American	75 (37.3%)
White	49 (24.4%)
More Than One Race	31 (15.4%)
Other	18 (9.0%)
Unknown Race	21 (10.4%)
Ethnicity	
Hispanic or Latino	96 (47.8%)
Not Hispanic or Latino	94 (46.8%)
Unknown Ethnicity	11 (5.5%)
State	
California	135 (67.2%)
Florida	66 (32.8%)
Current financial situation	
I cannot get by on the money I have	42 (22.1)
I can barely get by on the money I have	92 (48.4)
I have enough money to live comfortably	56 (29.5)
Prefer not to answer/ missing	11
Ever been unhoused, or lived in a shelter	
No	91 (50.3)
Yes	90 (49.7)
Prefer not to answer/ missing	20
Unmet subsistence score in the past 3 months	0.84 (0,1)
Mobile Technology Vulnerability Score in the past 6 months	0.06 (0,0.29)

^1^Gender Identity categories are not mutually exclusive.

IQR: interquartile range

Half of participants (48.4%, *n* = 92) reported that they currently could barely get by on the money they had, while 22.1% (*n* = 42) said they could not get by, and 29.5% (*n* = 456) said they had enough money to live comfortably (Table 1). The unmet subsistence needs score in the past 3 months had a median of 0.84 (IQR 0–1 out of a range of 0 to 5). The median MTVS score in the past 6 months was 0.06 (IQR 0,0.29; range 0–1). Nearly half (49.7%, *n* = 90) of participants had ever experienced housing instability, homelessness, or living in a shelter.

The majority of participants (89.1%; *n* = 179) of YWH were on oral ART, whereas 10.0% (*n* = 20) were receiving LAI-ART, and 1% (*n* = 2) were not currently taking ART (Table 2). Overall, 72.0% (*n* = 136) of participants were adherent to their ART regimen. Specifically, 69.8% (*n* = 118) of oral ART users reported adherence ≥ 80%, while 90% (*n* = 18) of LAI-ART users did not report missing or delaying their injections.

**Table 2:** HIV related outcome measures

	N (%)
Currently taking HIV medications	
No	2 (1.0)
Yes, injections	20 (10.0)
Yes, oral ART	179 (89.1)
Had an office visit with your HIV provider in the past 6 months	
No	18 (9.0)
Yes	183 (91.0)
How frequently was your injection delayed in the last 6 months?	
Not at all	18 (90.0)
A few times	1 (5.0)
More than half of the time	0 (0)
Nearly every time	0 (0)
Prefer not to answer/ missing	1 (5.0)
How frequently did you miss your injection altogether in the last 6 months?	
Not at all	19 (95.0)
A few times	0 (0)
More than half of the time	0 (0)
Nearly every time	0 (0)
Prefer not to answer/ missing	1 (5.0)
Adherent to oral ART (≥80%) or LAI (not missed or delayed)	
No	53 (28.0)
Yes	136 (72.0)
Missing/ not taking HIV medications	12
Adherent to oral ART only (≥80%)	
No	51 (30.2)
Yes	118 (69.8)
Missing/not taking oral ART	32
Adherent to LAI (not missed or delayed)	
No	2 (10.0)
Yes	18 (90.0)
Missing/not taking LAI	181

YWH who were non-adherent had more financial well-being challenges (Table 3). Participants who were classified as non-adherent were more likely to report that they could not get by on the money they had than those who were classified as adherent (37.3%, *n* = 19 versus 13.3%, *n* = 17, *p* =.001, χ²=16.23). YWH who were non-adherent reported a higher mean unmet subsistence score (mean 1.36, SD 1.62) than those who were adherent (mean 0.59, SD 1.28, *P* <.001, t = 3.44) and a higher mobile technology vulnerability score (mean 0.25, SD 0.24) than those who were adherent (mean 0.15, SD 0.22; *p* =.006, t = 2.78). YWH who were nonadherent were also more likely to have ever been unhoused or in unstable housing (60.4%, *n* = 29 versus 44.7%, *n* = 55; *p* =.065), although this difference was not statistically significant. Bivariate associations remained similar when examining ART adherence to oral ART alone versus to any ART (Table 3).

**Table 3:** Measures of financial well-being in relation to ART adherence outcomes

	Non-adherent	Adherent	*P*-Value	Test Statistic
	*N* (%)	*N* (%)		
Adherence to oral ART (≤80%) or LAI (not missed or delayed; *n* = 189)	(*n* = 53)	(*n* = 136)		
Ever been unhoused, or lived in a shelter			0.065	χ²=3.58
No	19 (39.6)	68 (55.3)		
Yes	29 (60.4)	55 (44.7)		
Prefer not to answer/ missing	5	13		
Current financial situation			0.001**	χ²=16.23
I cannot get by on the money I have	19 (37.3)	17 (13.3)		
I can barely get by on the money I have	24 (47.1)	64 (50.0)		
I have enough money to live comfortably	8 (15.7)	47 (36.7)		
Prefer not to answer	2	8		
Unmet subsistence score in the past 3 months, mean (SD)	1.36 (1.62)	0.59 (1.28)	< 0.001**	t = 3.44
Mobile Technology Vulnerability Score in the past 6 months, mean (SD)	0.25 (0.24)	0.15 (0.22)	0.006**	t = 2.78
Adherence to oral ART only (≥ 80%; *n* = 169)	(*n* = 51)	(*n* = 118)		
Ever been unhoused, or lived in a shelter			0.035*	χ²^=^ 5.82
No	19 (40.4)	63 (58.9)		
Yes	28 (59.6)	44 (41.1)		
Prefer not to answer/ missing	4	11		
Current financial situation			0.001**	χ²^=^17.12
I cannot get by on the money I have	18 (36.7)	13 (11.6)		
I can barely get by on the money I have	24 (49.0)	58 (51.8)		
I have enough money to live comfortably	7 (14.3)	41 (36.6)		
Prefer not to answer/ missing	2	6		
Unmet subsistence score in the past 3 months, median (IQR)	1.41 (1.63)	0.61 (1.29)	0.008**	t = 3.41
Mobile Technology Vulnerability Score in the past 6 months, mean (SD)	0.25 (0.24)	0.15 (0.21)	0.006**	t = 2.77

ART: antiretroviral therapy; IQR: interquartile range; SD: standard deviation; * *p* <.05, ** *p* <.01; χ²^=^ Chi^2^ Value; t = t-test

Table 4 shows crude and adjusted associations between measures of financial well-being and ART adherence. Compared to those who reported having enough money to live comfortably, participants who reported that they could barely get by on the money they had had a significantly lower prevalence of adherence (aPR 0.70; 95% CI 0.52–0.95). Those who could not get by had a lower prevalence of adherence, although this difference was not statistically significant (aRR 0.69; 95% CI 0.46–1.04). Unmet subsistence needs were also inversely associated with adherence, whereby participants with higher continuous unmet subsistence needs had lower adherence (aPR 0.85; 95% CI 0.73, 0.99). However, adherence was not significantly associated with housing stability or MTVS when adjusting for covariates (Table 4). When analyzing oral ART users separately, these associations remained unchanged.

**Table 4:** Prevalence ratio (PR) and 95% confidence intervals (CIs) for the association between measures of financial well-being and adherence

	Unadjusted PR (95% CI)	Adjusted PR* (95% CI)
Adherence to oral ART (≥80%) or LAI (not missed or delayed)		
Ever been unhoused, or lived in a shelter	0.84 (0.69, 1.01)	0.82 (0.65, 1.04)
Current financial Situation		
I cannot get by on the money I have	0.55 (0.38, 0.79)**	0.69 (0.46, 1.04)
I can barely get by on the money I have	0.85 (0.72, 1.01)	0.70 (0.52, 0.95)*
I have enough money to live comfortably	1	1
Unmet subsistence score in the past 3 months	0.88 (0.79,0.98)*	0.85 (0.73, 0.99)*
Technology vulnerability score in the past 6 months	0.55 (0.32, 0.93)*	0.47 (0.21, 1.03)
Adherence to oral ART (≥80%) only		
Ever been unhoused, or lived in a shelter	0.79 (0.64, 0.99)*	0.79 (0.60, 1.03)
Current financial Situation		
I cannot get by on the money I have	0.49 (0.32, 0.76)*	0.65 (0.41, 1.01)
I can barely get by on the money I have	0.83 (0.69, 0.99)**	0.70 (0.51, 0.97)*
I have enough money to live comfortably	1	1
Unmet subsistence score in the past 3 months	0.87 (0.77, 0.97)*	0.86 (0.74, 1.00)*
Technology vulnerability score in the past 6 months	0.50 (0.28,0.92)*	0.49 (0.22, 1.11)

*adjusting for age, sex at birth, race, ethnicity, and state

ART: antiretroviral therapy; CI: confidence interval; RR: risk ratio

* *p* <.05, ** *p* <.01

## Discussion

This study examined the relationship between financial well-being and ART adherence among 201 YWH in California and Florida, including those using LAI-ART and oral ART. Participants who reported financial instability and greater unmet subsistence needs were significantly less likely to be adherent to their ART regimen. This association remained significant even after adjusting for demographic factors, suggesting that financial hardship independently contributes to poor adherence. Our findings highlight the critical role of financial well-being in ART adherence among YWH.

Findings that financial instability and increased unmet subsistence needs decrease ART adherence align with previous research indicating that financial insecurity can create barriers to healthcare access, medication adherence, and retention in HIV care [13, 23–25]. Financial instability may contribute to difficulties in maintaining regular clinic appointments, affording transportation, and managing competing survival priorities, such as housing and food security. In our study, a substantial proportion of participants had experienced homelessness or shelter stays, reinforcing the link between structural vulnerabilities and health disparities. Our study underscores the need for comprehensive interventions that address financial barriers. Approaches such as financial assistance programs, transportation support, housing support, and food vouchers, combined with case management services, could help mitigate financial stressors and improve ART adherence.

Notably, adherence was high among participants using LAI-ART, although the sample size was small (*n* = 20). While LAI-ART has been recognized as a promising intervention to improve adherence by reducing the burden of daily medication-taking, its accessibility remains limited [15, 17]. Structural barriers, including inadequate insurance coverage, limited transportation options, and clinic accessibility challenges, may disproportionately affect youth facing financial hardship. Expanding access to LAI-ART at a low cost, particularly for financially vulnerable populations, could help bridge adherence gaps and improve virologic suppression in YWH.

While this study provides valuable insights, it has several limitations. First, the sample included youth who engaged in care within urban health settings, owned a smartphone, and spoke English. This may limit the study’s generalizability. Second, all measures relied on self-reports, which may introduce recall or social desirability biases. Financial well-being was also measured through self-report, which may not fully capture the multidimensional nature of financial instability, including access to public assistance, debt burden, or household financial dynamics. The measure of housing stability was also a lifetime measure and did not capture more recent housing instability, which may have been more likely to influence ART adherence. Third, the data were cross-sectional and therefore could not account for temporality or provide causal evidence. Fourth, the modest sample size (*n* = 201) may have reduced the power to examine relationships when adjusting for covariates. Additionally, the absence of objective adherence indicators (e.g., pharmacy refill records, viral load trajectories, medication event monitoring systems) limits the precision of adherence assessment. Finally, the study did not include broader structural determinants such as insurance instability, transportation barriers, state-level policies, or lifetime housing instability, which may confound or modify the association between financial well-being and ART adherence.

Future studies should use longitudinal designs to address temporal ordering and causality, incorporate objective adherence measures, and examine structural and policy-level factors to better capture the upstream conditions that affect financial well-being and ART adherence in YWH. In conclusion, financial well-being is a key determinant of ART adherence among YWH. Addressing financial barriers through targeted interventions and structural policy changes may be essential to improving adherence and overall health outcomes in this population. Expanding access to LAI-ART and providing financial support mechanisms could serve as critical strategies in ensuring sustained HIV care engagement and viral suppression among vulnerable youth.

## Data Availability

Data are available upon request by contacting the corresponding author.
